# Complete Genome Sequences of the *Potyvirus Sweet potato virus 2* from East Timor and Australia

**DOI:** 10.1128/genomeA.00504-16

**Published:** 2016-06-02

**Authors:** Solomon Maina, Owain R. Edwards, Luis de Almeida, Abel Ximenes, Roger A. C. Jones

**Affiliations:** a School of Plant Biology and Institute of Agriculture, Faculty of Science, The University of Western Australia, Crawley, Western Australia, Australia; b Cooperative Research Centre for Plant Biosecurity, Canberra, ACT, Australia; c CSIRO Entomology, Floreat Park, Western Australia, Australia; d Seeds of Life Project, Ministry Agriculture and Fisheries, Dili, East Timor; e DNQB-Plant Quarantine International Airport Nicolau Lobato Comoro, Dili, East Timor; f Department of Agriculture and Food Western Australia, South Perth, Western Australia, Australia

## Abstract

We present here the first complete genome sequences of *Sweet potato virus 2* (SPV2) from sweet potato in Australia and East Timor, and compare these with five complete SPV2 genome sequences from South Korea and one each from Spain and the United States. Both were closely related to SPV2 genomes from South Korea, Spain, and the United States.

## GENOME ANNOUNCEMENT

To examine possible connectivity between viruses infecting crops in Australia and Southeast Asia, we studied sweet potato viruses from East Timor and Australia. *Sweet potato virus 2* (SPV2; synonym, *Sweet potato virus Y*) is a single-stranded RNA virus within the genus *Potyvirus,* family *Potyviridae* (1). It was found previously in Australia, and six Australian coat protein sequences exist (AM050884 to AM050888 [1] and EF990648 [2]), but it is not reported from East Timor. Seven complete SPV2 genomes are available on GenBank, five from South Korea, and one each from Spain and the United States (3 – 5). Australian sample AusCan and sample Tm37 collected in May 2015 from the Dili district of East Timor were sequenced and their complete genomes obtained. AusCan and Tm37 were coinfected with *Sweet potato chlorotic fleck virus* (2, 6).

Fifteen East Timorese samples blotted onto Fast Technology for Analysis of nucleic acids (FTA) cards (7) were sent to Australia. A plant infected with AusCan (CP accession no. EF990648) was planted, and a scion from it was graft-inoculated to the indicator host *Ipomoea setosa*. Total RNA was extracted from the FTA cards, and from an *I. setosa* leaf sample with virus symptoms, using the ZR plant RNA MiniPrep kit (Zymo Research). The total RNA extracts were treated with RNase-free DNase (Invitrogen) and measured using Qubit (Invitrogen). RNA integrity was confirmed using RNA screen tape (TapeStation 2200; Agilent Technologies). Libraries were prepared from total RNA using a TruSeq stranded Total RNA sample preparation Ribo-Zero plant kit (catalog no. RS-122-2401; Illumina). The final size and concentration of each library were verified using Qubit and D1000 ScreenTape (TapeStation 2200; Agilent Technologies). Sequencing was performed by HiSeq 2500 using a TruSeq SBS kit version 4 (Illumina) with 151 cycles of paired-end reads. The reads were assembled and genomes annotated using CLC Genomics Workbench 6.5 (CLC bio) and Geneious 8.1.7 (Biomatters) (8).

FTA card sample Tm37 yielded 14,721,488 reads and, after trimming, 14,546,337 reads remained. *De novo* assembly generated 1,307 contigs and 13,415 reads mapped to the contig of interest, with a coverage of 298×. Sample AusCan yielded 6,260,728 reads, and, after trimming, 5,405,928 reads remained. *De novo* assembly generated 832 contigs, with 964,917 reads mapping to the contig of interest, giving a coverage of 1,460×. Both Tm37 and AusCan SPV2 sequences coded for 10 proteins, as occurs with other potyviruses (5, 9). The pairwise nucleotide sequence identity between Tm37 and AusCan was 97.7%, which is well within the species demarcation limit of 76% for complete *Potyvirus* genomes (10, 11). The closest pairwise identity for Tm37 was 98.7% to accession no. KP115617 from South Korea, and for AusCan, identity was 98.6% to accession no. KU511270 from Spain, followed by 98.3% to accession no. JN613807 from the United States. Thus, the Australian and East Timorese isolates were marginally less closely related to each other than to isolates from other parts of the world.

### Nucleotide sequence accession numbers.

The sequences were deposited in GenBank under accession numbers KX017447 (Tm37) and KX017448 (AusCan).

## References

[1] Tairo F, Jones RAC, Valkonen JPT 2006 Potyvirus complexes in sweet potato: occurrence in Australia, serological and molecular resolution, and analysis of the *Sweet potato virus 2* (SPV2) component. Plant Dis 90:1120–1128. doi:10.1094/PD-90-1120. 30781090

[2] Jones RAC, Dwyer GI 2007 Detection of *Sweet potato chlorotic fleck virus* and *Sweet potato feathery mottle virus*—strain O in Australia. Australas Plant Pathol 36:591–594. doi:10.1071/AP07069.

[3] Kwak H-R, Kim J, Kim M-K, Seo J-K, Jung M-N, Kim J-S, Lee S, Choi H-S 2015 Molecular characterization of five potyviruses infecting Korean sweet potatoes based on analyses of complete genome sequences. Plant Pathol J 31:388–401. doi:10.5423/PPJ.OA.04.2015.0072. 26673876 PMC4677748

[4] Mingot A, Valli A, Rodamilans B, San León D, Baulcombe DC, García JA, López-Moya JJ 2016 The P1N−PISPO trans-frame gene of sweet potato feathery mottle potyvirus is produced during virus infection and functions as an RNA silencing suppressor. J Virol 90:3543–3557. doi:10.1128/JVI.02360-15. 26792740 PMC4794657

[5] Li F, Xu D, Abad J, Li R 2012 Phylogenetic relationships of closely related potyviruses infecting sweet potato determined by genomic characterization of *Sweet potato virus G* and *Sweet potato virus 2*. Virus Genes 45:118–125. doi:10.1007/s11262-012-0749-2. 22562225

[6] Maina S, Edwards OR, de Almeida L, Ximenes A, Jones RAC 2016 Complete genome sequences of the *Carlavirus Sweet potato chlorotic fleck virus* from East Timor and Australia. Genome Announc 4(3):e00414-16. doi:10.1128/genomeA.00414-16. 27231359 PMC4882940

[7] Ndunguru J, Taylor NJ, Yadav J, Aly H, Legg JP, Aveling T, Thompson G, Fauquet CM 2005 Application of FTA technology for sampling, recovery and molecular characterization of viral pathogens and virus-derived transgenes from plant tissues. Virol J 2:45. doi:10.1186/1743-422X-2-45. 15904535 PMC1180858

[8] Kehoe MA, Coutts BA, Buirchell BJ, Jones RAC 2014 Plant virology and next generation sequencing: experiences with a *potyvirus*. PLoS One 9:e104580. doi:10.1371/journal.pone.0104580. 25102175 PMC4125191

[9] Revers F, García JA, Karl M, Thomas CM 2015 Molecular biology of potyviruses. Adv Virus Res 92:101–199. doi:10.1016/bs.aivir.2014.11.006. 25701887

[10] Adams MJ, Antoniw JF, Fauquet CM 2005 Molecular criteria for genus and species discrimination within the family *Potyviridae*. Arch Virol 150:459–479. doi:10.1007/s00705-004-0440-6. 15592889

[11] King AMQ, Adams MJ, Lefkowitz EJ 2011 Virus taxonomy: classification and nomenclature of viruses: ninth report of the International Committee on Taxonomy of Viruses, vol 9. Elsevier Academic Press, San Diego, CA.

